# Dual-energy CT iodine map in predicting the efficacy of neoadjuvant chemotherapy for hypopharyngeal carcinoma: a preliminary study

**DOI:** 10.1038/s41598-022-25828-5

**Published:** 2022-12-09

**Authors:** Xianfeng Wei, Rui Cao, Han Li, Miaomiao Long, Peipei Sun, Yongzhe Zheng, Li Li, Jianzhong Yin

**Affiliations:** 1 Department of Otolaryngology Head and Neck, Tianjin First Central Hospital; Key Laboratory of Auditory Speech and Balance Medicine; Key Medical Discipline of Tianjin (Otolaryngology); Otolaryngology Clinical Quality Control Centre, Tianjin Institute of Otolaryngology, Tianjin, 300192 China; 2 Medical Imaging Department, Tangshan Gongren Hospital, Tangshan, 063003 Hebei Province China; 3 The First Central Clinical College of Tianjin Medical University, Tianjin, 300070 China; 4 Department of Radiology, Tianjin First Central Hospital, Tianjin, 300192 China; 5 Department of Radiology, Haikou People’s Hospital, Affiliated Haikou Hospital of Xiangya Medical School, Central South University, Haikou, 570208 Hainan Province China

**Keywords:** Cancer therapy, Head and neck cancer, Oncology

## Abstract

Neoadjuvant chemotherapy has become one of the important means for advanced hypopharyngeal carcinoma. So far, there is no effective index to predict the curative effect. To investigate the value of iodine map of dual-energy computed tomography (CT) in predicting the efficacy of neoadjuvant chemotherapy for hypopharyngeal carcinoma. A total of 54 hypopharyngeal carcinomapatients who underwent two courses of TPF neoadjuvant chemotherapy were recruited in this study. Three cases had a complete response (CR), thirty-six cases had a partial response (PR), eleven cases had stable disease (SD), and four cases had a progressive disease (PD) after the chemotherapy. All patients underwent a dual-source CT scan before chemotherapy and rescanned after chemotherapy. The normalized iodine-related attenuation (NIRA) of the mean of maximum slice and most enhanced region of lesion at arterial and parenchymal phase were measured: NIRA_mean-A_, NIRA_max-A_, NIRA_mean-P_, and NIRA_max-P_, respectively. Correlation analysis was conducted between different metrics of NIRA and the diameter change rate of lesions, and the curative effect was evaluated based on the receiver operating characteristic (ROC) curve. There were a significant correlation between NIRA_mean-A_, NIRA_max-A_, NIRA_mean-P_, NIRA_max-P_ and the change rate of lesion’s maximum diameter (ΔD%) (all *P* < 0.01). The NIRA_max-A_, NIRA_mean-P_, NIRA_max-P_ had significant differences between CR, PR, SD, PD groups, but NIRA_mean-A_ did not reach a significant difference. All NIRA_mean-A_, NIRA_max-A_, NIRA_mean-P_, NIRA_max-P_ had significant differences between effective (CR + PR) and ineffective (SD + PD) groups. The ROC analysis revealed that NIRA_mean-P_ had the largest AUC and prediction efficacy (AUC = 0.809). Dual-energy CT iodine map could predict the efficacy of neoadjuvant chemotherapy and provides imaging evidence to assist in treatment decisions for hypopharyngeal carcinoma patients.

## Introduction

Hypopharyngeal carcinoma is relatively rare and accounts for about 3% of all head and neck cancers (HNCs). But it has a poor prognosis among all the HNCs with a 30–35% 5-year overall survival (OS) rate^1^. Around 80% of the patients were already at stages III and IV of the disease at the time of admission; only a few cases were detected at an early stage due to its concealed site and infiltrating growth. In recent years, a comprehensive treatment model of neoadjuvant chemotherapy, followed by concurrent radiotherapy and chemotherapy or surgery significantly improved the larynx preservation rate of locally advanced patients and improved the life quality of patients. Thus, predicting the efficacy of chemotherapy is crucial for the optimization of the treatment and avoiding unnecessary system toxicity, cost, and treatment delay. The evaluation of the efficacy of chemotherapy relies mainly on medical imaging. Dual-source dual-energy CT (DECT) could not only obtain single-energy images at different kV but also could generate iodine maps through data processing from different kV images, which might explicate the tissue characteristics of local hypopharyngeal carcinoma quantificationally^2^. And it was already reported that the enhanced CT iodine map could evaluate the histopathological invasiveness of lung cance^3^.

This study aimed to explore the predicting value of dual-source DECT in the evaluation of the efficacy of neoadjuvant chemotherapy for hypopharyngeal carcinoma.

## Materials and methods

### Patients

A total of 54 patients (50 males and 4 females, age range: 42 ~ 81 years old) were recruited in this study in Tianjin First Central Hospital from September 2014 to July 2019. All patients were pathologically confirmed to present hypopharyngeal squamous cell carcinomas, including 48 cases at the piriform fossa (88.9%), 5 cases (9.2%) at the posterior pharyngeal wall, and 1 case (1.9%) at the postcricoid region. All patients were given neoadjuvant chemotherapy with two courses of the modified TPF regimen (Docetaxel + Nedaplatin + Flurouracil). This study was consistent with the principles expressed in the Declaration of Helsinki and approved by the Ethics Committee of Tianjin First Central Hospital. The informed consent was waived for this retrospective study.

Inclusion criteria: (1) admitted to hospital due to hypopharyngeal neoplasms; (2) pathologically confirmed as hypopharyngeal carcinoma; (3) without any systematic treatment at the first diagnosis; (4) had no contraindications to chemotherapy and agreed to receive neoadjuvant chemotherapy; (5) underwent the dual-source DECT plain and enhanced scan before neoadjuvant chemotherapy, and rescanned within one week after the end of the second chemotherapy course.

Exclusion criteria: (1) poor image quality due to artifacts or other factors; (2) neoadjuvant chemotherapy was not performed on time because of complication, contraindication, or adverse reaction of chemotherapy; (3) less than two courses of neoadjuvant chemotherapy due to other reasons.

### Scan settings

The SOMATOM Definition Dual-source CT (DSCT; Siemens, Forchheim, Germany) was used to perform a routine plain and dual-energy enhanced scan. Patients lay in a prone position, breathed calmly, and were asked to not cough or swallow during CT scans. The plain scan was performed firstly, followed by an enhanced scan in dual-energy mode (tube voltage, 100 and 140 kV; tube current, Auto mA CARE Dose4D; pitch, 0.9; collimation, 64 × 0.6; reconstruct thickness, 3 mm). A mechanical high-pressure syringe was used to inject the iodine contrast agent (350 mgI/ml) through the elbow vein with a flow of 3 ml/s, dose 1 ~ 1.5 ml/kg and followed by 35 ml physiological saline. The dual-phase enhanced scan was performed at 30 ~ 45 s (arterial phase) and 80 ~ 90 s (parenchymal phase) after injection in energy mode.

### Image post-processing

The image data was transmitted to a Siemens workstation (Syngo MMwP 70,971) for post-processing. The fusion image of the anatomy and iodine map was obtained after loading to the Dual-Energy software. The maximum diameter of the tumor before and after chemotherapy was measured three times. The averaged diameter was recorded as D_Pre_ and D_Post_, and the change rate of maximum diameter was denoted as ΔD%.

The measurements of the iodine map were performed on the maximum slice of the lesion. The iodine-related attenuation (IRA) of the mean of the lesion and most enhanced region were measured three times. The averaged IRA of the whole lesion in this slice and the most enhanced region in the arterial phase and parenchymal phase were recorded as IRA_mean-A_, IRA_max-A_, IRA_mean-P_, and IRA_max-P_. Moreover, these metrics were normalized by the IRA value of the common carotid artery on the same side of the lesion and denoted as NIRA_mean-A_, NIRA_max-A_, NIRA_mean-P_, and NIRA_max-P_ in percentage, respectively. The ROIs of the most enhanced region were about 8mm^2^ in general and should include ten voxels at least for the minimum lesions, avoiding the gas, blood vessels, bones, cysts, and necrosis. The ROIs were measured by a 5-year-experience radiologist (R. C.) under the guidance of a radiologist (J. Y.) with over 20 years of experience.

### Evaluating metrics

1. Change rate of lesion’s maximum diameter (ΔD%) = (the tumor’s maximum diameter before chemotherapy (D_Pre_) − the tumor’s maximum diameter after chemotherapy (D_Post_))/the tumor’s maximum diameter before chemotherapy (D_Pre_) × 100%
2. Normalized iodine-related attenuation (NIRA) = iodine value of each period/iodine value of common carotid artery in the same period × 100%
3. According to Response Evaluation Criteria in Solid Tumors (RECIST), (1) complete response (CR): all lesions disappeared without any new lesion; (2) partial response (PR): the sum of the longest diameter of the measurable lesions is reduced by ≥ 30%, and no new lesions appear; (3) stable disease (SD): lesion decline is less than PR or lesion increase is less than that of the progressive disease; (4) progressive disease (PD): the longest diameter of the measurable lesions is increased by ≥ 20%, or new lesion appears. The CR + PR was considered effective while SD + PD was ineffective.

### Statistical analysis

Data analyses were conducted using SPSS25.0 software, and the correlation analysis used the Spearman correlation method. Independent-sample Kruskal–Wallis test and independent-sample Mann–Whitney U test was used for enumeration data, and *P* < 0.05 indicated a statistically significant difference. The receiver operating characteristics curve (ROC) was plotted for curative effect evaluation, and the sensitivity, specificity, area under the curve (AUC), and optimal cutoff value were calculated. A combination of AUC > 0.5 and *P* < 0.05 in the ROC curve indicated a significant difference.

## Results

### General information of patients

**Figure 1:**
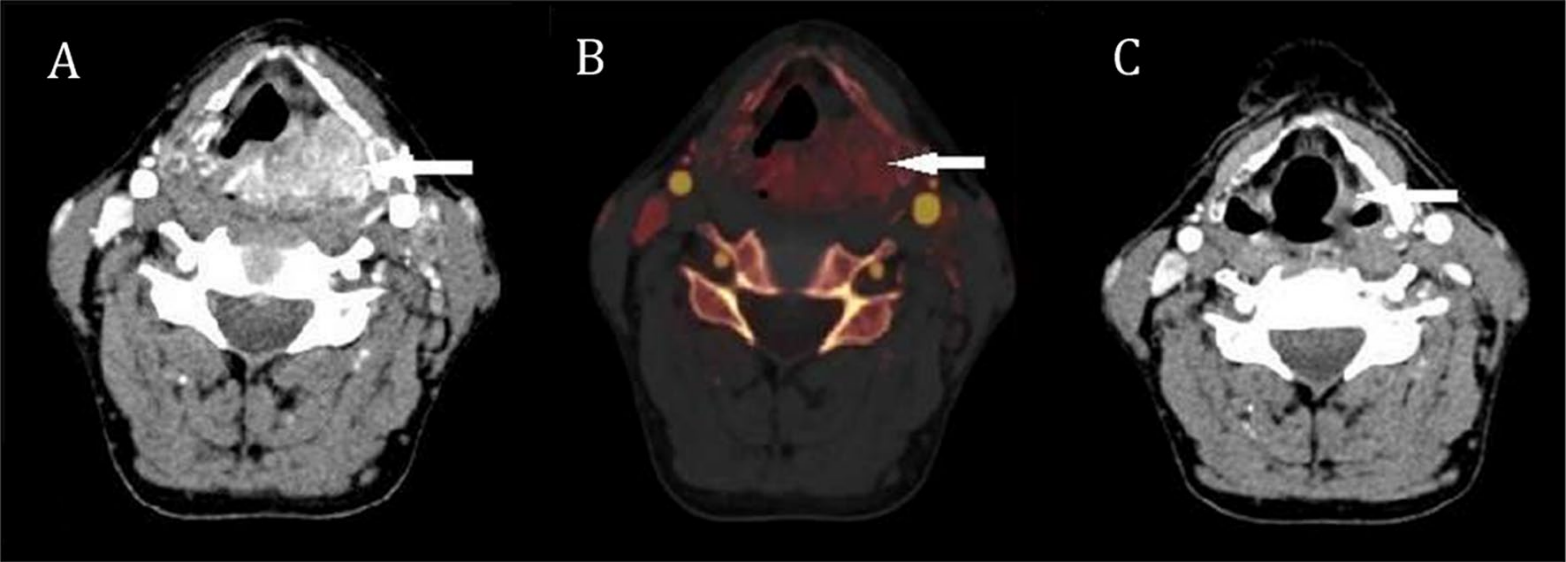
A representative hypopharyngeal cancer patient showed partial remission after neoadjuvant chemotherapy. (**A**) Axial CT image of arterial phase before chemotherapy showed an obviously enhanced tumor at left piriform fossa extending to the posterior pharynx wall and postcricoid region. (**B**) The iodine map of arterial phase before chemotherapy, the red color showed obvious iodine-related attenuation (IRA_mean_ 48.8HU, IRA_max_ 76.5HU) in the tumor. (**C**) Followup CT image after chemotherapy, the lesion at the left piriform fossa was significantly smaller.

**Figure 2:**
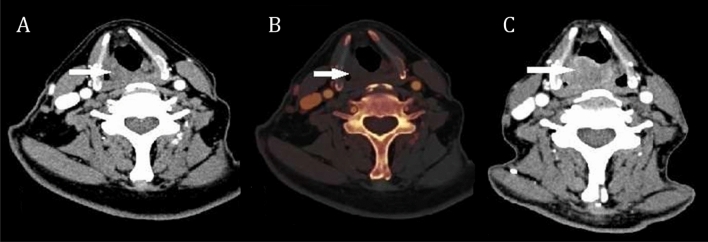
A representative patient with progressive hypopharyngeal cancer. (**A**) Axial CT image of arterial phase before chemotherapy, showed a tumor at the right piriform fossa but no obviouly enhancement. (**B**) The iodine map of arterial phase before chemotherapy, there was no obvious color in the lesion which implied a low iodine-related attenuation (IRA_mean_ 16.7HU, IRA_max_ 22.1HU). (**C**) Followup CT image after chemotherapy, showed that the tumor at right piriform fossa was significantly enlarged.

All 54 patients, including 3 cases of CR, 36 cases of PR, 11 cases of SD, and 4 cases of PD, completed neoadjuvant chemotherapy and underwent CT scans as required. These patients were classified into effective and ineffective groups (n = 39 and 15, respectively). The information about the patients and the diameter changes of the lesions were summarized in Table 1. Figure 1 showed a 66-year-old male patient with left piriform fossa carcinoma who had a partial response after chemotherapy. Figure 2 showed a 70-year-old male patient with right piriform fossa progressive carcinoma.

**Table 1:** Information of patients and diameter changes of the lesions.

	Effective		Ineffective		Total
	CR	PR	SD	PD	
Age (years)	64.67 ± 15.57	60.89 ± 9.16	66.27 ± 9.57	67.50 ± 5.80	62.69 ± 9.52
Male/Female	3/0	34/2	10/1	3/1	50/4
D_Pre_	2.50 ± 0.94	3.11 ± 0.97	3.07 ± 1.36	2.59 ± 0.48	3.03 ± 1.02
D_Post_	0.00 ± 0.00	1.11 ± 0.63	2.49 ± 1.06	3.65 ± 0.72	1.52 ± 1.13
ΔD%	1.00 ± 0.00	0.63 ± 0.17	0.19 ± 0.12	− 0.42 ± 0.27	0.48 ± 0.37

*CR* complete response; *PR* partial response; *SD* stable disease; *PD* progressive disease.

### Normalized iodine-related attenuation (NIRA) and response evaluation (RECIST)

**Figure 3:**
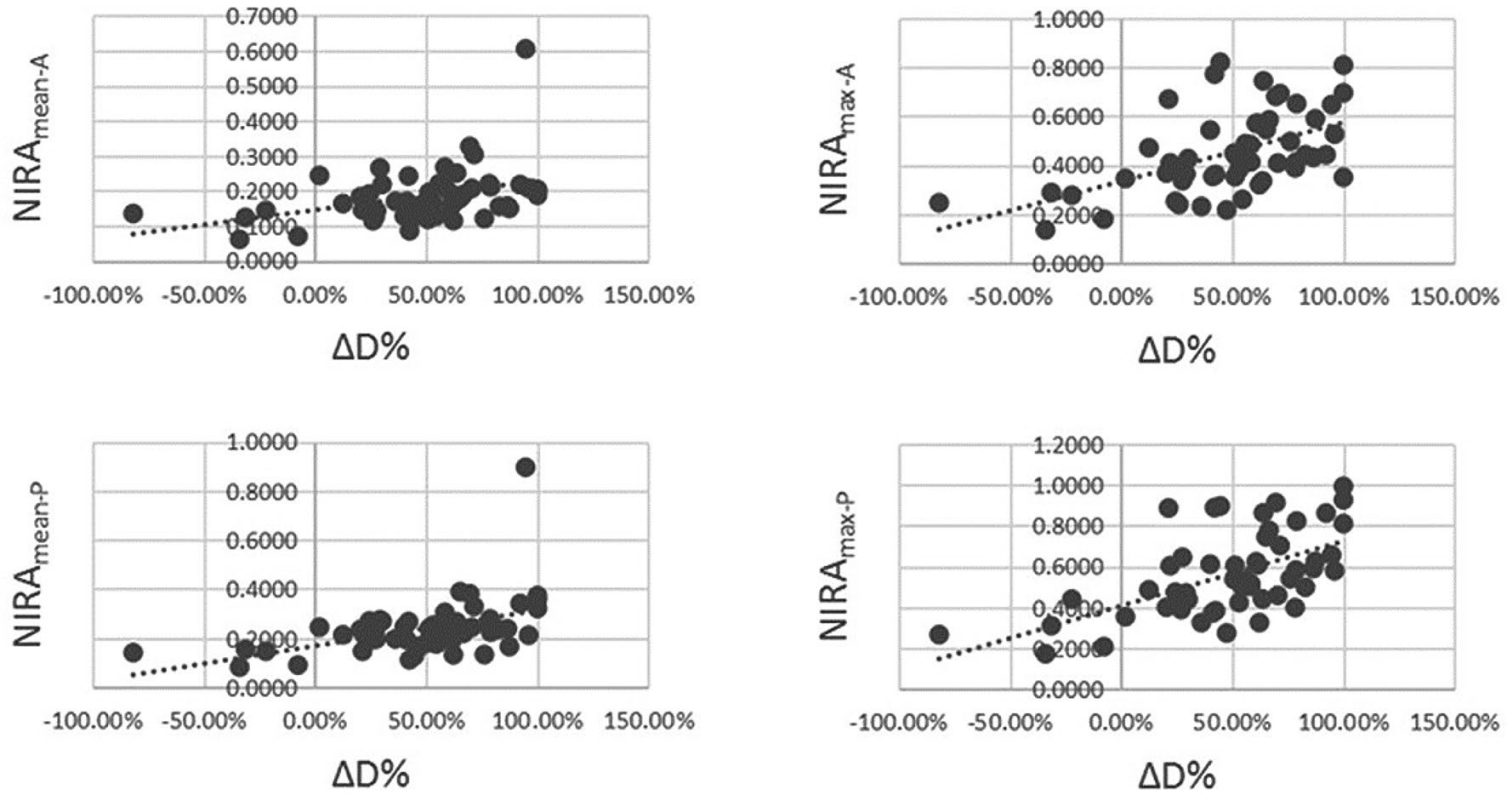
Scatter plot to illustrate the correlation between normalized iodine-related attenuation (NIRA) and the change rate of lesion’s maximum diameter (ΔD%) (Spearman analysis, all *P* < 0.01).

Spearman analysis revealed there was a significant correlation between NIRA_mean-A_, NIRA_max-A_, NIRA_mean-P_, NIRA_max-P_ and the change rate of the lesion’s maximum diameter (ΔD%) (all *P* < 0.01, Fig. 3).

**Figure 4:**
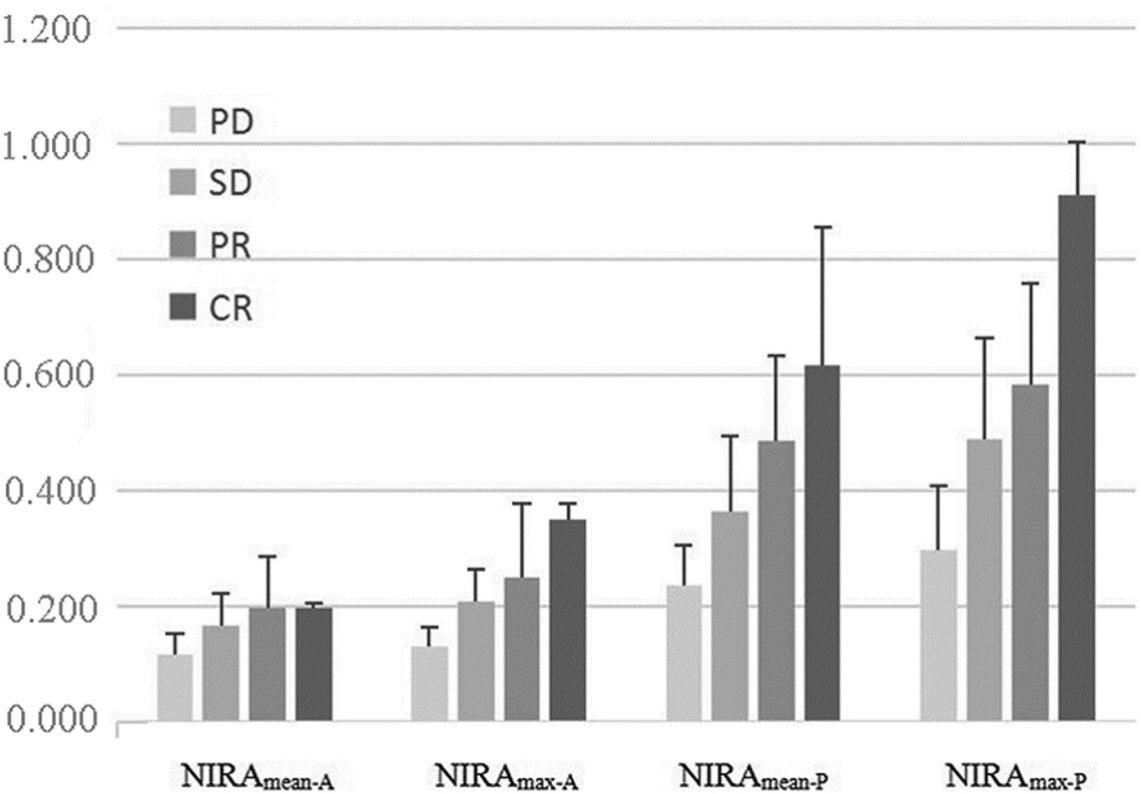
Histogram of the NIRA_mean-A_, NIRA_max-A_, NIRA_mean-P_, NIRA_max-P_ between CR, PR, SD, PD groups. NIRA_max-A_, NIRA_mean-P_, NIRA_max-P_ had significant differences but NIRA_mean-A_ did not reach a significant difference between different postchemotherapy response groups.

Further Kruskal–Wallis test on the NIRA_mean-A_, NIRA_max-A_, NIRA_mean-P_, and NIRA_max-P_ between different postchemotherapy response groups showed that NIRA_max-A_, NIRA_mean-P_, NIRA_max-P_ had significant differences between CR, PR, SD, PD groups (all *P* < 0.01), but NIRA_mean-A_ did not reach a significant difference (*P* = 0.05) (Fig. 4 and Table 2).

**Table 2:** Comparison of NIRAs among PD, SD, PR, and CR groups.

Groups	NIRA_mean-A_	NIRA_max-A_	NIRA_mean-P_	NIRA_max-P_
CR	0.197 ± 0.009	0.351 ± 0.027	0.618 ± 0.238	0.911 ± 0.092
PR	0.198 ± 0.087	0.249 ± 0.129	0.485 ± 0.149	0.583 ± 0.174
SD	0.166 ± 0.056	0.210 ± 0.054	0.364 ± 0.130	0.488 ± 0.177
PD	0.116 ± 0.037	0.131 ± 0.033	0.237 ± 0.070	0.299 ± 0.111
*P* value	0.050	0.004	0.002	0.001
*Power*	0.403	0.473	0.857	0.890

*NIRA* Normalized iodine-related attenuation; *NIRA*_*mean-A*_ the mean NIRA of maximum slice of lesion at arterial phase; *NIRA*_*max-A*_ the NIRA of most enhanced region of lesion at arterial phase; *NIRA*_*mean-P*_ the mean NIRA of maximum slice of lesion at parenchymal phase; *NIRA*_*max-P*_ the NIRA of most enhanced region of lesion at parenchymal phase; *CR* complete response; *PR* partial response; *SD* stable disease; *PD* progressive disease.

To evaluate the efficacy of neoadjuvant chemotherapy, all the cases were divided into an effective group (CR + PR) and an ineffective group (SD + PD). And Mann–Whitney U test revealed that all NIRA_mean-A_, NIRA_max-A_, NIRA_mean-P_, and NIRA_max-P_ had significant differences between effective and ineffective groups (Table 3).

**Table 3:** Comparison of NIRAs between effective and ineffective groups.

Groups	NIRA_mean-A_	NIRA_max-A_	NIRA_mean-P_	NIRA_max-P_
Ineffective	0.153 ± 0.055	0.189 ± 0.060	0.330 ± 0.129	0.438 ± 0.180
Effective	0.198 ± 0.084	0.257 ± 0.127	0.496 ± 0.157	0.609 ± 0.190
*P* value	0.022	0.030	< 0.001	0.004
*Power*	0.450	0.490	0.895	0.755

*NIRA* Normalized iodine-related attenuation; *NIRA*_*mean-A*_ the mean NIRA of maximum slice of lesion at arterial phase; *NIRA*_*max-A*_ the NIRA of most enhanced region of lesion at arterial phase; *NIRA*_*mean-P*_ the mean NIRA of maximum slice of lesion at parenchymal phase; *NIRA*_*max-P*_ the NIRA of most enhanced region of lesion at parenchymal phase.

### ROC analysis and predicting the efficacy of neoadjuvant chemotherapy

**Figure 5:**
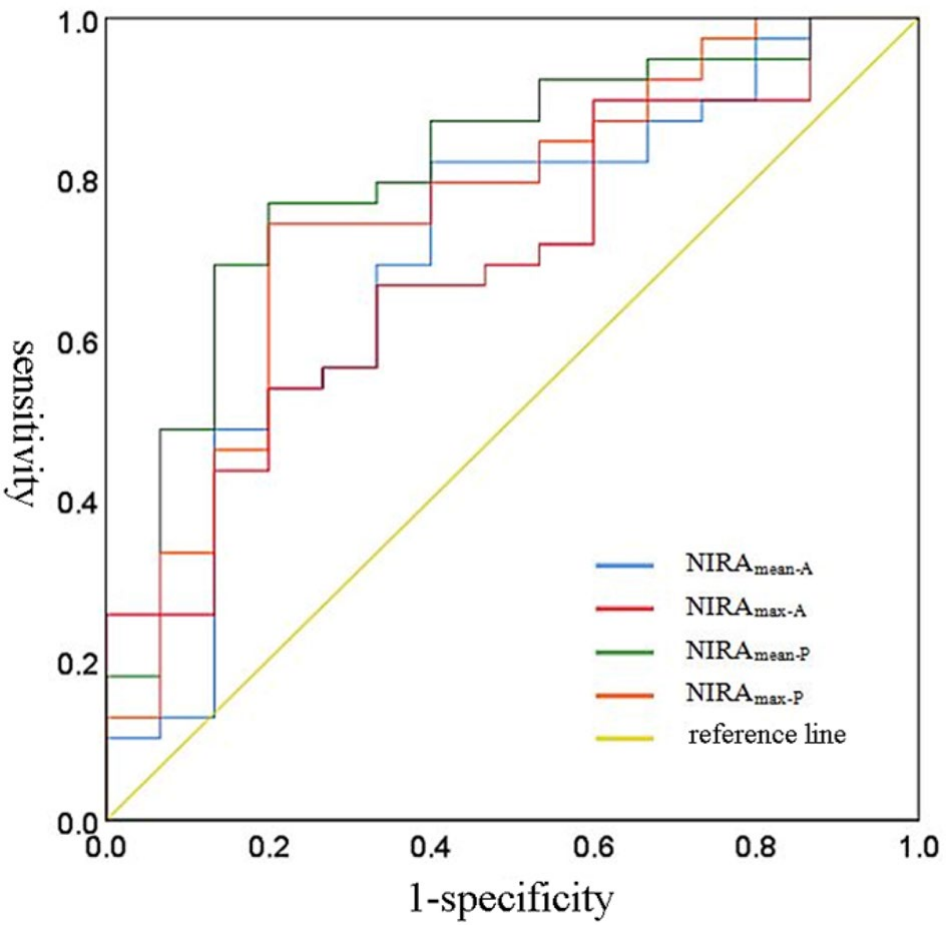
ROC analysis NIRA_mean-A_, NIRA_max-A_, NIRA_mean-P_, NIRA_max-P_ to predict the efficacy of neoadjuvant chemotherapy.

The ROC analysis revealed that all NIRA_mean-A_, NIRA_max-A_, NIRA_mean-P_, and NIRA_max-P_ could be used to predict the efficacy of neoadjuvant chemotherapy (AUC > 0.5, *P* < 0.05). The NIRA_mean-P_ had the largest AUC and prediction efficacy (AUC = 0.809) (Fig. 5 and Table 4). For the different parametric of NIRA, the mean NIRA of the maximum slice showed a larger AUC and Youden index than the most enhanced region of the lesion at the same time phase. The NIRA at the parenchymal phase had a larger area under the ROC curve and predicting specificity than the arterial phase, but the NIRA_mean-A_ had a higher sensitivity. The best cutoff values of NIRA_mean-A_, NIRA_max-A_, NIRA_mean-P_, and NIRA_max-P_ were 0.148, 0.236, 0.375, and 0.494, respectively (Table 4).

**Table 4:** The ability of different NIRA parameters in predicting the efficacy of neoadjuvant chemotherapy.

	AUC	*P*	Cut-off value	Sensitivity	Specificity	Youden index
NIRA_mean-A_	0.703	0.022	0.148	0.821	0.600	0.421
NIRA_max-A_	0.692	0.030	0.236	0.538	0.800	0.338
NIRA_mean-P_	0.809	< 0.001	0.375	0.769	0.800	0.569
NIRA_max-P_	0.757	0.004	0.494	0.744	0.800	0.544

*AUC* area under the curve; *NIRA* Normalized iodine-related attenuation; *NIRA*_*mean-A*_ the mean NIRA of maximum slice of lesion at arterial phase; *NIRA*_*max-A*_ the NIRA of most enhanced region of lesion at arterial phase; *NIRA*_*mean-P*_ the mean NIRA of maximum slice of lesion at parenchymal phase;* NIRA*_*max-P*_ the NIRA of most enhanced region of lesion at parenchymal phase.

## Discussion

Hypopharyngeal cancer is a severe malignancy with a poor prognosis and mortality rate. Neoadjuvant chemotherapy is one of the major methods of comprehensive treatment of head and neck squamous cell carcinoma and has been advocated in recent years with satisfactory results^4^. The evaluation of the efficacy of chemotherapy as early as possible has an important significance for the treatment and prognosis of patients. Various functional techniques, including positron emission tomography (PET) or magnetic resonance imaging (MRI) diffusion-weighted imaging^5,6^ or dynamic contrast-enhanced MRI^5,7^, and CT perfusion^8,9^ were employed to evaluate the metabolism, hypoxia, cellularity, and perfusion of the tumor. Nonetheless, some drawbacks, such as high cost, long scanning time, and poor resolution of small lesions, limit the clinical application of these methods. As new technology emerged in recent years, dual-source DECT imaging assesses the degree of tumor vascularization^10^. The applications of DECT in head and neck carcinoma (HNC) has increased, and several studies have shown the benefit of iodine characterization and virtual monoenergetic images for the detection and delineation of HNC^11,12^, differentiation between metastatic, inflammatory, and benign cervical lymph nodes^13–15^, or assessment of cartilage invasion^16–18^. However, the role of DECT-derived quantitative imaging to predict oncological outcomes in hypopharyngeal squamous cell carcinoma has not yet been investigated.

The images obtained in this study were used to evaluate the efficacy based on the morphological changes of RECIST^19^; however, the internal tumor variation, such as liquefaction necrosis, is unable to make an effective assessment due to these limitations^20,21^. The RECIST standard assesses the curative effect based on the change in the maximum tumor diameter, which might include tumor and necrotic areas, while the modified RECIST (mRECIST) standard uses “surviving tumors” as the evaluation criteria, excluding the affect of necrotics, recommended by 2010 EASL(European Association for the Study of the Liver) guidelines^22^. Dual-energy CT can obtain a series of derivative images, including iodine map, virtual non-enhanced image, single energy spectrum image, and nonlinear fusion image. Among these, the iodine-related attenuation measured in the iodine map quantificationally reflects the distribution and uptake of iodine in the lesion to reveal its blood supply condition. Moreover, the iodine-related attenuation is more reliable than the density value if the lesion is enhanced, for example the iodine-related attenuation is not affected by intratumoral hemorrhage. The content of iodine in the tissue can reflect its blood supply, and our results showed it might be a promising biological marker for evaluating the efficacy of chemotherapy. In this study, the patients with hypopharyngeal carcinoma with higher iodine-related attenuation were sensitive to neoadjuvant chemotherapy. Zima et al.^23^ reported that HNC with high perfusion were sensitive to radiotherapy and chemotherapy, which is consistent with the current conclusion. Thus, it could be postulated that the high perfusion state of tumor tissue can concentrate the local drug than the low perfusion state, thereby promoting the cell killing effect. The hypoperfusion state of tumor tissue might be considered as tumor ischemia and hypoxia, inducing the insensitivity to radiotherapy and chemotherapy.

Neoadjuvant chemotherapy is one of the comprehensive treatment measures for advanced hypopharyngeal cancer. In 1991, the Laryngeal Cancer Group of American Veterans discovered the laryngeal function-sparing effects of chemotherapy and radiotherapy^24^. In 2003, the US RTOG 91–11 study confirmed that the laryngeal function-sparing rates of concurrent radiotherapy and chemotherapy (CRC), radiotherapy (RT), and neoadjuvant chemotherapy + radiotherapy (IC + RT) were 84%, 66%, and 71%, respectively^25^. Currently, non-surgical treatments, including CRC, IC + RT, and RT combined with EGFR^4^ are applied to preserve function in laryngeal and hypopharyngeal cancer patients. However, some patients not sensitive to chemotherapy should need surgery. So that evaluate the effectiveness of the chemotherapy and adjust the treatment plan in time are critical to the survival and prognosis of the patient. Currently, there is no effective method to predict the curative effect before neoadjuvant chemotherapy. This study revealed that Dual-energy CT iodine map could be used to screen out the cases might have favorable effects before chemotherapy and avoid the inappropriate treatment plan for ineffective cases.

The RECIST morphological standard was as the reference to evaluate the tumor curative effect objectively. In order to eliminate the influence of the injection rate and dose of contrast agent among different individuals, a normalized iodine-related attenuation (NIRA) was applied in this study, which is the ratio of the iodine-related attenuation (IRA) in the ROI to the IRA in the carotid artery of the same layer. The present study showed that the NIRAs before chemotherapy was significantly related to the change of tumor diameter, including NIRA_mean-A_, NIRA_max-A_, NIRA_mean-P_, and NIRA_max-P_. For the different parametrics of NIRA, the NIRA at parenchymal phase had a larger area under the ROC curve and predicting specificity than the arterial phase, but the NIRA_mean-A_ had a higher sensitivity. In addition to measuring the mean NIRA of maximum slice of lesion (NIRA_mean_), we also measured the NIRA of most enhanced region of lesion (NIRA_max_) at the arterial and parenchymal phases which might avoid the influence of necrosis within the tumor. But the results showed that NIRA_mean_ had a better predicting effect than NIRA_max_, implied that NIRA_mean_ might be better to reflect the global characteristic of tumor. The ROC curve reflects the correlation between the sensitivity and specificity of different NIRA parametrics, thereby rendering it as a comprehensive indicator of the test’s accuracy^26^. Other researches also confirmed that vascular iodine concentration (IC) reflects the efficacy of chemotherapy drugs within the tissue^27–31^. Yang et al.^32^ revealed that patients with higher HU values had a significantly low risk of progression and local recurrence, and DECT could easily identify CR patients and aid in choosing the appropriate treatment regimen for advanced laryngeal and hypopharyngeal squamous cell carcinoma (LHSCC). Bahig et al.^33^ reported that maximum IC of the primary tumor and the high volume and IC standard deviation of involved lymph nodes predict the local regional recurrence in LHSCC. Therefore, DECT provides valuable information to evaluate the response of hypopharyngeal carcinoma which might be important for the clinical implementation of individualized treatment. In addition, Ge et al.^34^ compared the radiation dose of DECT and conventional CT scan mode and did not find any significant difference in the mean radiation dose between DECT and the conventional CT scan, indicating that DECT did not cause additional radiation damage and was safe and suitable for efficacy evaluation.

Nevertheless, the present study has several limitations. The evaluation was only for short-term efficacy, and the reliability of the results for the long-term prognosis requires further verification with large sample data. Using dual-source CT iodine-related measurement to predict the effect of chemotherapy is a universal law based on correlation, and its accuracy and feasibility need to be assessed for individual cases. Moreover, the accuracy of iodine-related attenuation measurement is hindered by several factors, such as the inhomogeneity of local structure, the algorithms, and ROI selection. But the progress and improvement of DECT would promote its clinical applications.

## Conclusions

In summary, our study showed that DECT iodine-related attenuation might be useful in clinical practice as a tool to stratify patients into appropriate treatment of neoadjuvant chemotherapy.

## Data Availability

The datasets used and/or analysed during the current study are available from the corresponding author on reasonable request.
